# Research into biomarkers to facilitate the early identification of Parkinson's disease: a decision analytic model to determine the feasibility and value

**DOI:** 10.1186/1745-6215-14-S1-P52

**Published:** 2013-11-29

**Authors:** Rachael Hunter, Gordon Proctor, Alastair Noyce, Anette Schrag, Elaine Ward, John Galloway

**Affiliations:** 1 University College London, London, UK; 2 King's College London, London, UK; 3 Central and North West London NHS Foundation Trust, London, UK; 4 UCLH NHS Foundation Trust, London, UK

## Introduction

Parkinson's disease (PD) is a neurodegenerative associated with a significant, negative impact on quality of life, health care and societal costs . As the condition progresses the burden of disease in PD increases. Earlier identification of PD could provide the opportunity to intervene earlier, with evidence that this can delay the onset of the disease. Currently though an early identification test for PD does not exist. The aim of this work is to establish the feasibility and value of identifying biomarkers in PD which could form the basis of an early identification test.

## Method

A decision analytic model was developed to determine the lower threshold of sensitivity and specificity that the test would require to be cost-effective compared to current practice. The model simulates the incidence and progression of PD over 10 years, with earlier identification and treatment stalling disease progress. Extensive probabilistic and deterministic sensitivity analysis was conducted as well as an analysis of the extra value of perfect information (EVPI).

## Results

For a willingness to pay for a quality adjusted life year (QALY) gained of £30,000, the new test would need to have minimum sensitivity of 0.8 and a specificity of 0.7. At these values there was a 52% probability that the test is cost-effective. Given the potential benefits and the significant uncertainty in the model, the EVPI suggests that investment into further research in this area could be of value.

## Conclusion

A test for the early detection of PD that is cost-effective appears to be feasible and worth the additional investment required.

